# Improvements of Sound Localization Abilities by the Facial Ruff of the Barn Owl (*Tyto alba*) as Demonstrated by Virtual Ruff Removal

**DOI:** 10.1371/journal.pone.0007721

**Published:** 2009-11-05

**Authors:** Laura Hausmann, Mark von Campenhausen, Frank Endler, Martin Singheiser, Hermann Wagner

**Affiliations:** Institute for Biology II, RWTH Aachen, Aachen, Germany; Rutgers University, United States of America

## Abstract

**Background:**

When sound arrives at the eardrum it has already been filtered by the body, head, and outer ear. This process is mathematically described by the head-related transfer functions (HRTFs), which are characteristic for the spatial position of a sound source and for the individual ear. HRTFs in the barn owl (*Tyto alba*) are also shaped by the facial ruff, a specialization that alters interaural time differences (ITD), interaural intensity differences (ILD), and the frequency spectrum of the incoming sound to improve sound localization. Here we created novel stimuli to simulate the removal of the barn owl's ruff in a virtual acoustic environment, thus creating a situation similar to passive listening in other animals, and used these stimuli in behavioral tests.

**Methodology/Principal Findings:**

HRTFs were recorded from an owl before and after removal of the ruff feathers. Normal and ruff-removed conditions were created by filtering broadband noise with the HRTFs. Under normal virtual conditions, no differences in azimuthal head-turning behavior between individualized and non-individualized HRTFs were observed. The owls were able to respond differently to stimuli from the back than to stimuli from the front having the same ITD. By contrast, such a discrimination was not possible after the virtual removal of the ruff. Elevational head-turn angles were (slightly) smaller with non-individualized than with individualized HRTFs. The removal of the ruff resulted in a large decrease in elevational head-turning amplitudes.

**Conclusions/Significance:**

The facial ruff a) improves azimuthal sound localization by increasing the ITD range and b) improves elevational sound localization in the frontal field by introducing a shift of iso–ILD lines out of the midsagittal plane, which causes ILDs to increase with increasing stimulus elevation. The changes at the behavioral level could be related to the changes in the binaural physical parameters that occurred after the virtual removal of the ruff. These data provide new insights into the function of external hearing structures and open up the possibility to apply the results on autonomous agents, creation of virtual auditory environments for humans, or in hearing aids.

## Introduction

The barn owl (*Tyto alba*) is an effective nocturnal hunter that has developed a unique morphological specialization, the directionally sensitive facial ruff [1]. While it seems clear that the ruff plays a role in prey capture and sound localization [2], its behavioral relevance is poorly understood at a quantitative level.

Barn owls localize sound by making saccadic head-turns towards the sound emitting source [3]. The contribution of auditory cues to azimuthal and elevational sound localization was investigated by stimulating both ears with earphones [4] or, more advanced, in a virtual acoustic space [5], [6], [7]. These experiments identified the interaural time difference (ITD) as the only cue that determines the amplitude of the azimuthal head-turn [6], [8], [9], [10], [11]. Interaural level differences (ILDs) were found to be an important cue for elevational sound localization [5], [7], [8], [9], [12]. ITD and ILD are processed independently in separate neural pathways [13]. Further cues, like the monaural spectra may help to resolve ambiguities, for example if ITD and ILD have identical values at several positions in space [14,15, see also 16].

Since the body, head and outer ear (facial ruff of the owl) influence ITDs, ILDs and the monaural characteristics of sounds arriving at the eardrum in a direction-dependent and frequency-specific manner, recording of the so-called head-related transfer functions (HRTFs) and convolution of any free-field sound with the appropriate HRTF for a given spatial position creates virtual acoustic stimuli (VAS). Presentation of VAS via earphones allows for externalization of sounds in humans (for a review see [23]). Poganiatz et al. [6] showed that barn owls responded to VAS in the same way as they responded to free-field sounds. Since the barn owl can barely move its eyes or ear flaps, the amplitudes of the head saccades can be used as a direct measure for the perceived sound-source position.

A full set of HRTFs in the barn owl was first measured by Keller et al. [5]. Recently, Campenhausen and Wagner [16] quantified the physical changes of sound-localization parameters like ITD and ILD, based on HRTFs recorded before and after removing the ruff feathers. After removal of the ruff, the ITD range was decreased (Figure S1D) and ILDs did no longer change with elevational stimulus position in the frontal hemisphere (Figure S1H). The VAS derived from the HRTFs may be manipulated for example by shifting ITDs [6] or by altering the correlation of binaurally presented noise [7]. These manipulations allow current studies to go beyond earlier studies [2]. We made use of these possibilities to virtually remove the ruff. This has the advantage that the ruff does not need to be cut off, which might influence the birds' behavior and would create an instable situation due to regrowth of feathers. Another advantage of VAS is that HRTFs from one individual may be used in the same (individualized HRTF), but also in other individuals (non-individualized HRTFs). This allows for a better generalization of the effects of stimulus parameters.

We here address the question to what extent the ruff influences azimuthal and elevational sound localization, and whether its function is accurately reflected by the changes that it introduces to the ITD and ILD distributions.

## Results

Experiments were carried out with three tame barn owls from the institute's breeding stock. Our hypothesis was that ruff removal influences sound localization and that the effect of ruff removal can be related to changes in the distributions of ITDs and ILDs. To test this hypothesis, it was first important to determine the distributions of ITDs and ILDs on an individualized (“normal, individualized condition” or stimulus condition 1) and non-individualized basis (“normal, non-individualized condition” or stimulus condition 2), then to show that non-individualized HRTFs are adequate for stimulation by comparing the behavioral responses to stimulus condition 2 with those to stimulus condition 1. Finally, we quantified how non-individualized HRTFs with removed ruff (“ruffcut condition” or stimulus condition 3) influence sound localization and relate the changes in sound localization induced by ruff removal to the accompanying changes in sound-localization parameters.

### Patterns of ITDs and ILDs in the HRTFs

Since we described the characteristics in ITD and ILD distributions before and after ruff removal, respectively, in an earlier study [16], we only present a short summary here. ITDs changed continuously with azimuth up to about 110° and were largely independent of elevation. The most prominent feature of the ITD distribution was a shift of the extrema of the ITD to the rear hemisphere (minimum ITD at about −110° azimuth/−20° elevation, maximum ITD at about +110° azimuth/+20° elevation; Figure S1A, S1B, S1C). The most eye-catching feature of the ILD distribution in the barn owl with its asymmetrical ears was a rotation of the axis with the largest ILD gradient from the azimuthal axis, resulting in a minimum of the ILD at about −20° azimuth/−20° elevation and a maximum of the ILD at about +20° azimuth/+20° elevation (Figure S1E, S1F, S1G). ILD is unambiguously related to varying elevational sound positions in an area spanning about +40° in elevation and about +60° in azimuth. At more peripheral positions (>±100° azimuth), ILD does not change with stimulus elevation and should, therefore, not be a reliable cue for elevation. Only in the frontal field (within approximately ±60° where auditory resolution is highest [17]), both sound source azimuth and elevation are unambiguously coded by a specific combination of ITD and ILD under normal conditions [18], [19], [20].

**Figure 1:**
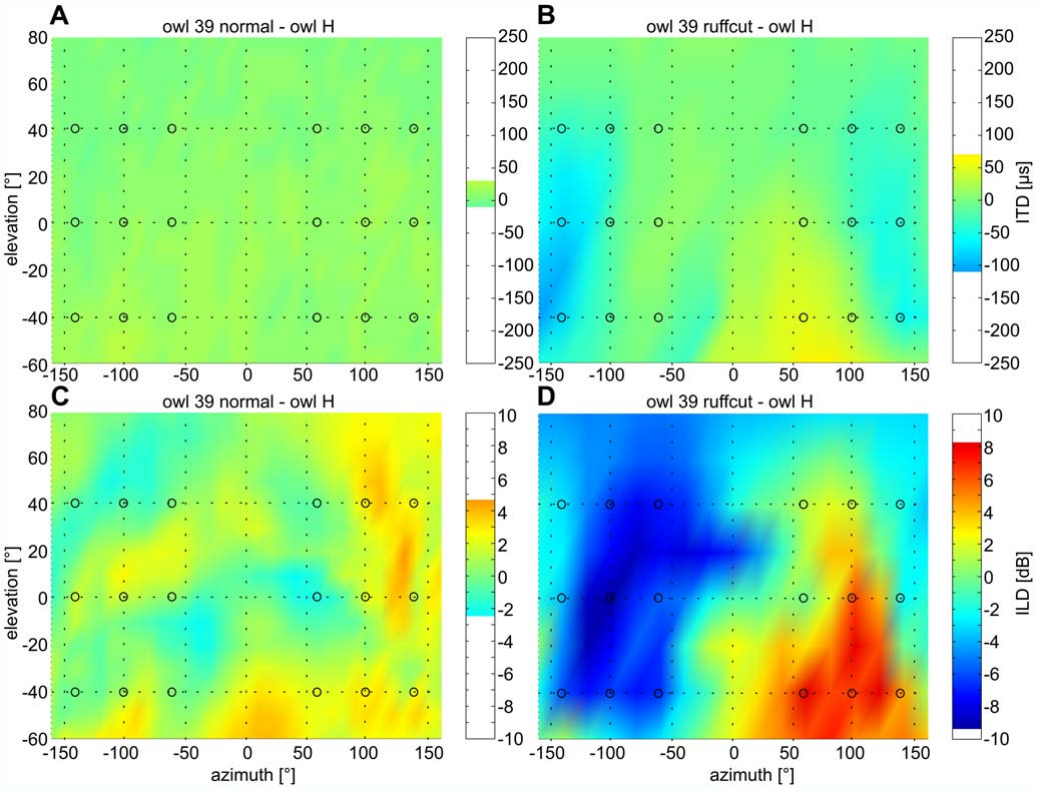
Differences between HRTFs of owl H and owl 39. The differences between the ITDs (upper panels) are shown for owl H and owl 39 with (A) intact and (B) removed ruff, respectively. Equivalent plots for ILD differences (lower panels) between owl H and owl 39 with (C) intact and (D) removed ruff feathers, respectively. Coloration is explained in the bar plots on the side. Blue areas mark positions where the ITDs or ILDs of owl 39 are smaller (nearer to 0 µs or dB) than those of owl H, red areas mean that the ITDs or ILDs of owl 39 are larger (>0 µs or dB). The differences in both, ITDs and ILDs, are larger when the facial ruff was removed than if it was intact (compare A to B and C to D).

Qualitatively, the distributions of ITDs and ILDs were similar between owls, but slight differences occurred. For example, in owl S, the main ILD extrema were shifted towards approximately ±135° azimuth and ±10° elevation, but there were local extrema at the positions indicated above (Figure S1F). We quantified the differences between the varying conditions by subtracting the corresponding ITDs and ILDs, respectively. The data of owl 39 with intact facial ruff served as reference. The normal individualized ITD and ILD distributions from the experimental owls H, P and S were compared with those of owl 39 (Figure 1A: ITDs, Figure 1C: ILDs, exemplary for owl H). For example, ITD differences between owl H and owl 39 normal ranged from −10 to +30 µs (Figure 1A). ILD differences ranged from about −2 to +5 dB (Figure 1C). The differences were only slightly higher for owls S (−50 to +30 µs ITD and −3 to +8 dB ILD, data not shown). Owl P's HRTFs differed from those of owl H by up to ±40 µs and −9 to +1 dB, and from those of owl 39 normal by −30 to 50 µs ITD and −2 to −8.5 dB ILD, respectively (data not shown). Hence, under normal conditions, the patterns of ITDs and ILDs were similar in all owls.

The distributions of ITDs and ILDs changed dramatically when the ruff was removed (compare Figure S1A, S1B, S1C to Figure S1D for ITDs, and to Figure S1H for ILDs). The most important effect of ruff removal on the distribution of ITDs was a shift of the ITD extrema from the rear hemisphere towards ±90° azimuth and 0° elevation, as is the case in species with symmetrical ears (Figure S1D). The ITD range decreased from about ±270 µs to about ±240 µs. After the ruff was removed, the characteristic kidney-shaped distribution of the iso-ILD lines (Figure S1E, S1F, S1G) was lost. ILDs in the ruffcut condition changed with azimuthal sound source position, but did no longer vary with elevation (Figure S1H). The quantitative analysis demonstrated differences in ITD of up to −110 to +70 µs and up to −9 to +8 dB in ILD, respectively (Figure 1B and 1D for owl H) between the individualized HRTFs and those of owl 39 after ruff removal. The differences between owl 39 (ruff removed) and owls P and S, respectively, were similar (−110 to +60 µs ITD for owl P and −90 to +40 µs ITD for owl S; −14 to 6 dB ILD for owl P and −7 to +8 dB ILD for owl S, data not shown). The largest differences occurred at the peripheral positions (beyond ±100° azimuth) and in the lower hemisphere. The spatial positions for stimulation of the owls lay within the areas with large differences in ITD and ILD. These were ±60°, ±100° and ±140° azimuth at ±40° and 0° elevation (marked with circles in Figure 1).

### Head-turns and response latencies

The owls were stimulated with broadband noise in a virtual acoustic space. The noise bursts were filtered with different sets of HRTFs, resulting in the three stimulus conditions. Two owls (owls H and S) were stimulated with their own (individualized) HRTFs or stimulus condition 1. All three owls were tested with stimulus condition 2, with HRTFs recorded from a reference owl with intact ruff (owl 39 normal). The third owl, P, was also stimulated with the HRTFs from owl H. The use of two sets of normal, non-individualized HRTFs in this owl served as a control condition to reveal possible learning effects, such as habituation to non-individualized stimuli which might influence the owl's performance. All three owls were also tested with stimulus condition 3. Comparison of the latter two stimulus paradigms reveals the contribution of the facial ruff for azimuthal and elevational sound localization.

**Figure 2:**
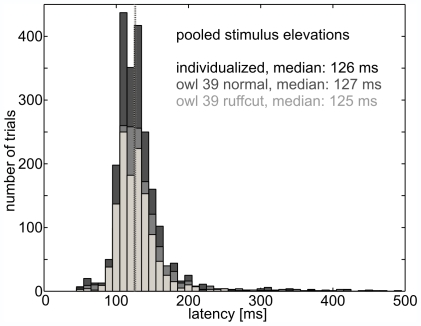
Latencies. Pooled latencies for the owls and stimulus positions are similarly distributed for trials using individualized HRTFs (dark gray, median at 125 ms marked by dark gray line), owl 39 normal (medium gray, median at 125 ms) and owl 39 ruffcut (light gray, median at 127 ms). Trials with latencies larger than 500 ms were excluded from the analysis, because they indicated low motivation or other distracters. No systematic effect of ruff removal on response latency was observed.

In all three stimulus conditions the owls responded to HRTF-filtered stimuli from varying azimuths (−140° to 140°) and elevations (±40° and 0°) with a saccadic head-turn into the stimulus direction (Figure S2). If the stimulation angle was negative, the owl turned its head to the left side. If the stimulus angle was positive, it turned the head to the right side. Head-turn latency was the time between stimulus onset and the first time point at which the head-turn velocity exceeded 20°/s (circle, Figure S2B). Head-turn latencies were concentrated between 80 and 200 ms for all owls (Figure 2) with medians ranging from 104 to 160 ms depending on the owl and stimulus position (see also Figure S3). These findings accord with previous latency measurements in the barn owl [3], [6], [21]. In general, latencies did not significantly differ between trials with non-individualized or individualized HRTFs at a particular stimulus position (Wilcoxon signed ranks test, two-tailed, p>0.05, Figure S3). Furthermore, the resemblance of latencies in all stimulus conditions was a first indication that the owls perceived all stimuli similarly.

### Azimuthal head-turns

**Figure 3:**
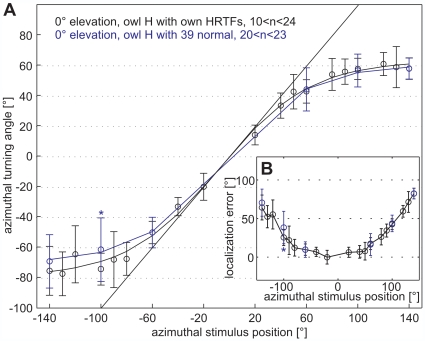
Azimuthal head-turn angles. (A) Head-turn angles in degree (mean±SD) are plotted against the azimuthal-stimulus position in degree, exemplary for owl H with individualized HRTFs at 0° elevation (black) and for stimulation with HRTFs of owl 39 with intact ruff (blue). Localization of the exact stimulus position would result in a line with a slope of 1 (black straight line). The curved black and blue lines are Boltzman fits (see Data analysis) to the azimuthal head-turn angles. Head-turn angles differed only at -100° azimuth (blue asterisk, Mann-Whitney test, p<0.05) between the two stimulus conditions (for the other owls, see Figure S4). The ranges of the number of trials (n) per day point are indicated. (B) The azimuthal-localization error (difference between stimulus angle and head-turn angle) is plotted as a function of the azimuthal-stimulus angle. Responses to individualized HRTFs are shown in black, those to owl 39 normal in blue. Localization errors were smaller for small stimulus angles than for large stimulus angles, which are reflected by an increasing localization error with increasingly peripheral stimulus angles (see also Figure S4).

The mean amplitude of the azimuthal head-turn saccade depended on the stimulus position in an unambiguous way as shown for owl H in Figure 3A. For both negative and positive stimulus azimuths, respectively, the absolute value of the amplitude increased in a monotonic way with the absolute value of the stimulus position (Figure 3A). Consequently, a sigmoidal Boltzman function (see Data analysis) fitted the data well for all owls (R^2^>0.9935 for individualized and R^2^>0.9889 for normal non-individualized HRTFs). This observation meant specifically that the owls were able to respond differently to stimuli coming from the rear hemisphere and to stimuli coming from the frontal hemisphere, even if these stimuli had the same ITD. However, at any azimuthal stimulus position, the amplitude of the head-turn was too small; in other words, the owls undershot the target position. The difference between the owl's head-turn angle and the target angle reflects the azimuthal localization error. For stimulus angles beyond ±100°, the amplitude of the azimuthal head-turns approached a plateau of about ±60° (Figure 3A). The effect of the elevation was significant (p<0.023, two-way ANOVA) for each of the three owls, as were the interaction terms of elevation and azimuth (p<0.001), respectively elevation and stimulus condition (owl H: p = 0.036, owl S: p = 0.029; owl P: p = 0.062).

Due to the increase of the undershooting with stimulus azimuth, the localization error exhibited a U-shaped dependence on azimuth (Figure 3B for owl H at 0° elevation; for the other owls and stimulus elevations see Figure S4). The elevation of the stimulus influenced the amplitude of the azimuthal head-turn (Figure S5). For both elevational positions of −40° and +40°, the mean azimuthal head-turn amplitude was reduced compared to the situation when stimulus elevation was 0° (Mann-Whitney test, p<0.05, Figure S5).

**Figure 4:**
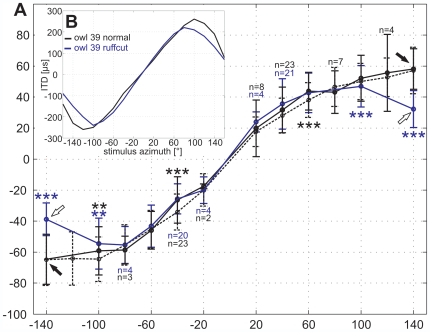
ITDs and azimuthal head-turn angle. (A) The azimuthal head-turn angles were pooled for stimulation with individualized HRTFs (dotted, owls H and S), responses to owl 39 normal (black, all three owls) and to owl 39 ruffcut (blue, all three owls). Stepsize was 20°. Arrows mark ±140° stimulus position, where the azimuthal head-turn angle decreased highly significantly (Mann-Whitney test, p<0.0001) in the ruffcut condition. Note that, in contrast to the ruffcut condition, the head-turn angles with intact ruff (individualized and owl 39 normal) approach a plateau at about ±60°. Significant differences between stimulus conditions are marked with asterisks depending on the significance level (**p<0.01, ***p<0.001) in black (individualized versus owl 39 normal) respectively in blue (owl 39 normal versus owl 39 ruffcut). Each data point includes at least 96 trials, unless indicated otherwise by the number of trials (n). (B) The ITD in µs contained in the HRTFs at 0° elevation is plotted against stimulus azimuth in degree for owl 39 normal (black) and owl 39 ruffcut (blue). Note the sinusoidal course of the ITD. The ITD decreased at peripheral azimuths for both intact as well as for removed ruff. The ITD range was smaller in the ruffcut condition.

The azimuthal localization error varied as much between the owls as it did between individualized stimuli (owls H and S) and owl 39 normal (Table 1). We compared responses at 59 positions (see owls H and S in Figure S4) and found differences with a Mann-Whitney test (p<0.05) only at 12 positions (20%, asterisks in Figure S4). A difference in the azimuthal turning behavior of owl P when stimulated with the two sets of non-individualized HRTFs was only observed in 1 of 31 tests (3.2%, Figure S4, Table 1). To compare the results obtained with the three stimulus conditions, we pooled the azimuthal head-turn angles for all owls (Figure 4A, stimulus condition 1: dotted; stimulus condition 2: black; stimulus condition 3: blue). Differences between conditions 1 and 2 occurred only at a few positions as indicated by the black asterisks in Figure 4A. The general relationship between stimulus angle and mean head-turn angle, as exemplary described above (Figure 3), was highly similar for all stimulus conditions with intact ruff. Altogether, these results demonstrated no systematic azimuthal head-turn differences between normal non-individualized HRTFs and individualized HRTFs.

**Table 1:** Differences between individualized and non-individualized HRTFs.

owl 39 normal	owl H individualized	owl S individualized	owl P owl H's HRTFs	owls H and S, pooled
**≤±60°, frontal**	9/11 (81.8%)	11/14 (78.6%)	13/13 (100%)	20/25 (80.0%)
**±100°, middle**	4/6 (66.7%)	6/6 (100.0%)	6/6 (100.0%)	10/12 (83.3%)
**±140°, peripher**	5/6 (83.3%)	6/6 (100.0%)	5/6 (83.3%)	11/12 (91.6%)
**owl 39 ruffcut**				
**≤±60°, frontal**	5/8 (62.5%)	9/10 (90.0%)	8/8 (100%)	14/18 (77.7%)
**±100°, middle**	1/6 (16.7%)	6/6 (100%)	5/6 (83.3%)	7/12 (58.3%)
**±140°, peripher**	0/6 (0.0%)	0/6 (0.0%)	0/6 (0.0%)	0/12 (0.0%)

We tested for each owl and stimulus azimuth whether the head-turn angles differed between HRTFs with intact ruff (owls H and S: individualized; owl P: HRTFs of owl H) and HRTFs of owl 39 normal, respectively owl 39 ruffcut (Wilcoxon signed ranks test, p<0.05). The frontal field (≤±60° stimulus azimuth), a position in the middle field (±100°) and in the periphery (±140°) were regarded separately. Differing positions are also marked with blue asterisks (owl 39 normal) and red asterisks (owl 39 ruffcut) in Figure S4. The first number in each column gives the number of pairings where the mean turning angles were not significantly different; the second number is the total number of tested pairings. The percentage is given in brackets. There were highly significantly (Fisher test, p<0.001) more differences at ±140° in the owl 39 ruffcut condition than in the owl 39 normal condition, but not in the frontal field.

On the other hand, the removal of the ruff had an influence on peripheral sound localization (stimulus angles outside ±60°). Changes in localization behavior occurred at positions where the differences in ITDs between intact and removed ruff were largest (Figure 1B). For all owls and stimulus elevations, the angular extent of the azimuthal head-turns decreased highly significantly for stimuli originating at ±100° azimuth (Figure 4A) and even stronger for stimuli corresponding to ±140° stimulus azimuth (Figure 4A, blue, and Figure S4). For example, when the stimulus was computed from an HRTF corresponding to ±140° in the ruffcut condition, the head-turns had amplitudes that corresponded to those measured for a stimulation from approximately ±40° in the ruff-intact condition (Table 2). The change in the amplitude of the head-turns was correlated with the decreasing ITDs in the periphery (see also next paragraph). By contrast, localization behavior generally did not differ between the three stimulus conditions within the frontal area of ±60° and only partly at ±100° stimulus angle (Figure 4A, Table 1, and Table 3). This finding was related to the small differences in ITDs within this area between the three stimulus conditions (Figure 1B). A two-way ANOVA with a Scheffé post-hoc test showed that the stimulus condition had a highly significant (p<0.001) influence on the azimuthal head-turn behavior in dependence of the azimuthal stimulus angle (Table 3). At ±140° stimulus angle, the head-turn angle was highly significantly shorter in the ruffcut condition than in any stimulus condition with intact ruff for all three owls, irrespective of whether the HRTFs were individualized or non-individualized (Table 3), whereas the differences between conditions with intact ruff was not significant (Table 3).

**Table 2:** Head-turn angles at peripheral stimulus positions.

		**owl H individual**		**owl H with 39n**		**owl H with 39c**	
**ele**	**azi**	**mean [°]**	**SD**	**mean [°]**	**SD**	**mean [°]**	**SD**
**40°**	**−140**	−60.31	10.98	−76.07	16.30	−39.68	6.72
	**140**	53.75	10.79	55.79	9.52	25.68	10.07
**0°**	**−140**	−75.71	16.15	−69.36	18.00	−44.62	9.66
	**140**	57.62	7.15	57.57	7.03	33.71	10.04
**−40°**	**−140**	−67.21	11.39	−66.55	16.52	−41.44	11.56
	**140**	57.19	10.95	57.57	13.16	38.50	8.27
		**owl S individual**		**owl S with 39n**		**owl S with 39c**	
	**azi**	**mean [°]**	**SD**	**mean [°]**	**SD**	**mean [°]**	**SD**
**40°**	**−140**	−56.92	16.47	−67.35	12.61	−35.51	8.81
	**140**	45.86	14.83	43.83	11.53	27.91	7.95
**0°**	**−140**	−66.43	14.75	−71.25	11.45	−44.10	13.17
	**140**	59.35	21.34	61.65	11.88	38.40	16.94
**−40°**	**−140**	−57.21	20.24	−52.39	12.45	−41.55	9.81
	**140**	62.35	11.53	61.69	13.97	33.79	10.62
		**owl P with owl H**		**owl P with 39n**		**owl P with 39c**	
	**azi**	**mean [°]**	**SD**	**mean [°]**	**SD**	**mean [°]**	**SD**
**40°**	**−140**	−47.63	9.36	−59.06	15.77	−31.23	8.76
	**140**	55.87	9.70	56.86	13.36	25.53	7.01
**0°**	**−140**	−64.93	10.83	−58.87	9.87	−37.43	10.40
	**140**	64.92	13.11	61.59	17.55	41.27	14.48
**−40°**	**−140**	−60.28	13.14	−55.19	12.49	−35.54	8.09
	**140**	64.57	17.97	59.10	12.37	31.67	12.32

For each owl and stimulus condition (39n = owl 39 normal, 39c = owl 39 ruffcut), the mean azimuthal head-turn angles±standard deviation (SD) at ±140° stimulus azimuth (azi) is given for the three stimulus elevations (ele). Responses to HRTFs of owl 39 ruffcut were highly significantly (Mann-Whitney test, p<0.001) smaller than responses to either individualized (respectively owl H's HRTFs in case of owl P) or owl 39 normal stimuli for any owl and elevation.

**Table 3:** ANOVA and Scheffé post-hoc test for azimuthal head-turn angles.

Azimuth			
**Owl H**	**Ind./39n**	**39n/39c**	**Ind./39c**
**−140**	0.459	0.000	0.000
**−100**	0.742	0.946	0.559
**−60**	0.077	0.283	0.785
**60**	0.016	0.997	0.009
**100**	0.556	0.173	0.702
**140**	0.873	0.000	0.000
**Owl S**	**Ind./39n**	**39n/39c**	**Ind./39c**
**−140**	0.413	0.000	0.000
**−100**	0.635	0.103	0.012
**−60**	0.777	0.322	0.118
**60**	0.049	0.023	0.931
**100**	0.848	0.003	0.015
**140**	0.971	0.000	0.000
**Owl P**	**Owl H/39n**	**39n/39c**	**Owl H/39c**
**−140**	0.775	0.000	0.000
**−100**	0.974	0.036	0.072
**−60**	0.031	0.629	0.226
**60**	0.863	0.566	0.276
**100**	0.762	0.175	0.028
**140**	0.607	0.000	0.000

For the three owls, a two-way ANOVA showed a highly significant (p<0.001) influence of the HRTF set on the azimuthal head-turn angle. A Scheffé post-hoc test whose results (p values) are given for each owl, azimuth and stimulus condition (ind. = individualized, 39n = owl 39 normal, 39c = owl 39 ruffcut) revealed that the difference was due to differences at peripheral stimulus angles (±140°), whereas responses to more central stimulus angles mostly did not differ.

**Figure 5:**
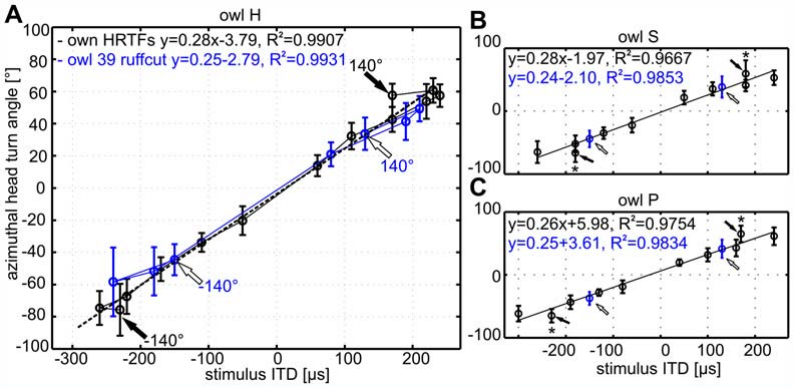
Prediction of azimuthal head-turns from the ITD. The ITD contained at 0° stimulus elevation in the HRTFs of (A) owl H, (B) owl S, and (C) owl P were plotted against the azimuthal head-turn angle in degree. A linear regression (dotted line) through all head-turn angles shows that the owls responded well to the ITD in the HRTFs within the frontal field (≤±60°). Linear equations and goodness of fit (R^2^) of the regression are stated in each panel. With individualized HRTFs at ±140° (black arrows), however, the head-turn angles significantly deviated from the regression line (Mann-Whitney test, p<0.05).

To further analyze the effect of ruff removal, we plotted the azimuthal head-turn angle as a function of ITD (Figure 5) for the normal condition (intact ruff, black line) and the ruff removed condition (blue line). We reasoned that all head-turn angles should lie on one line, if the owl localized targets in both the frontal and rear hemisphere based on ITD alone. The data points for all stimulus angles up to ±100° fulfilled this expectation. By contrast, a significant deviation from the regression line was observed for stimulus azimuths of ±140° in the normal condition (black arrows and asterisks in Figure 5, one-sample t-test, p<0.05; p<0.08 for owl H at −140°). Thus, under normal conditions, the owls associated different positions in space to targets having the same ITD, but originating from different azimuths. This is only possible, if the owls used further cues to distinguish between targets with the same ITD. Without the ruff these additional cues apparently cannot be used, since the azimuthal head-turn angle did not deviate from the responses obtained with frontal-hemisphere stimulation in this condition (white arrows in Figure 5). Thus, in the ruffcut condition the owls behaved as if they used the ITD as the exclusive cue for stimulus azimuth.

### Elevational head-turns

**Figure 6:**
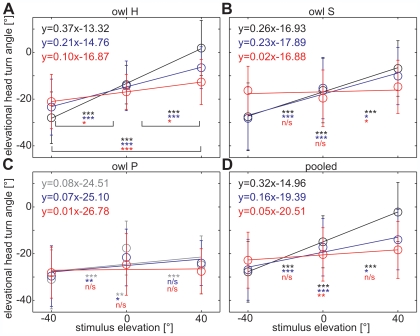
Elevational head-turn angles. The elevational head-turn angles (mean±SD) are plotted against stimulus angles for (A–C) owls H, S, and P individually as well as (D) the pooled data from all owls. Since the owls reacted to stimulus elevation even with individualized HRTFs only in the frontal area, stimulus angles of <±60° were included. With individualized HRTFs (A,B, black line and circles), there was a significant increase in the elevational turning angle with stimulus elevation (Mann-Whitney test, p<0.001). The slope of this increase was lower, but still significant with non-individualized HRTFs of owl 39 normal (blue). In the ruffcut condition (red), the slope was significantly different from 0 only for owl H, but neither for owl S nor for owl P. Owl P reacted similar to non-individualized HRTFs (gray: owl H's HRTFs, blue: owl 39 normal) as the other two owls, but located stimuli at 40° elevation lower than those at 0° elevation. However, the general characteristics of the elevational head-turn behavior were preserved in that the increase of head-turn angle with stimulus elevation was strongly reduced in the ruffcut condition for all owls compared to HRTFs recorded with intact ruff (D). The linear equations are given for each stimulus condition. For each pair of stimulus angles (−40° and 0°, 0° and 40°, and −40° and 40°), head-turn angles were compared with a Mann-Whitney test; significant differences are marked with asterisks (***p<0.001, **p<0.01, *p<0.05). Each data point includes at least 18 trials.

The owls were stimulated at elevations of −40°, 0° and 40° from various azimuthal positions. The amplitude of the elevational head-turn component was positively correlated with stimulus elevation for stimulus positions within a frontal area of about ±60° azimuth, when individualized HRTFs were used (Figure 6A and 6B). For stimulus azimuths outside the frontal area the elevational head-turn amplitude was not correlated with stimulus elevation. This is paralleled by a less clear relation between ILD and elevation outside the frontal area (Figure S1E, S1F, S1G). In the following, we focus on the frontal area to investigate the effect of ruff removal on elevational sound localization.

Responses to non-individualized stimuli often resulted in smaller mean elevational head-turn amplitudes than responses to individualized stimuli (Figure 6A and 6B). For example, whereas owl H had a mean turning angle of −28° when it was tested with its own HRTFs at −40° elevation, the mean turning angle was −23.4° when this owl was tested with non-individualized HRTFs of owl 39 normal (Figure 6A). This difference was significant in a t-test (p<0.01). Similar observations were made with a stimulus elevation of +40° and with the other owls, which resulted in smaller slopes of the relationship between stimulus elevation and elevational head-turn angle when individualized HRTFs were compared to non-individualized HRTFs with intact ruff (Figure 6A: owl H, Figure 6B: owl S). Owl P responded similar to the other owls for stimulus elevations −40° and 0° (Figure 6C). For a stimulus elevation of 40°, however, this owl located stimuli lower than those at 0° elevation (Figure 6C).

These results demonstrated that in elevation, the owls responded to normal non-individualized HRTFs slightly different than to individualized HRTFs. Differences in ILDs between individualized and normal non-individualized HRTFs were up to 8.5 dB (see above and Figure 1C) or about 40% of the normal range (compare with Figure S1), depending on the stimulus position.

The ILD differences were increased between the stimulus condition 3 and stimulus condition 1. In the ruffcut condition, owl H was still able to discriminate the three stimulus elevations (Figure 6A), whereas owl S distinguished only stimuli at 0° from those at 40° elevation, but did not discriminate between these two elevations and −40° elevation (Figure 6B, red asterisks). For owl P, no significant differences in the elevational head-turn angles were found in the ruffcut condition for any stimulus elevation (Figure 6C).

To compare the different dependencies of the elevational head-turn amplitudes on the varying stimulus conditions, the data were pooled (Figure 6D). The slope in the ruffcut condition (Figure 6D, red, pooled for all owls) was clearly smaller than the slopes in the normal, individualized condition (black, pooled for owls H and S) and the non-individualized condition with intact ruff (blue, pooled for all owls). This indicated that the facial ruff provides information that helps the owl to improve localization of elevational target positions.

**Figure 7:**
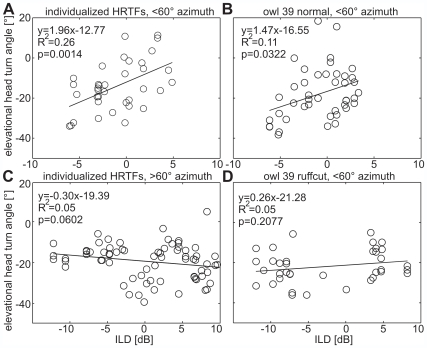
Elevational localization related to ILDs. For the frontal field (±60°), elevational head-turn angles were plotted against the ILDs in (A) individualized and (B) non-individualized HRTFs with intact ruff. Both factors were significantly correlated at the indicated level (p) within the frontal field, where ILDs strongly varied with elevation (see Figure S4). (C) At more peripheral positions, where ILDs did no longer vary with elevation, ILDs and elevational head-turn angles were not correlated. (D) The same held for the ruffcut condition, where ILDs were not correlated with the elevational head-turn angle neither in the frontal field (D), nor in the periphery (data not shown).

To test whether the changes in elevational localization were due to changes of the ILD distributions, the ILDs in the HRTFs were correlated with the amplitudes of the elevational head-turns (Figure 7). A significant correlation existed for both individualized HRTFs (owls H and S, Figure 7A) and normal non-individualized HRTFs (Figure 7B) within the frontal area of ±60° where ILDs strongly varied with stimulus elevation (Figure S1E, S1F, S1G). At more peripheral positions where ILDs varied less systematically with elevation, ILDs were not correlated with the elevational head-turn angles (Figure 7C). In the ruffcut condition ILDs and elevational head-turn angle were not correlated even within the frontal field (Figure 7D). This is consistent with the observation that ILDs did not vary with elevation in this condition (Figure S1H).

## Discussion

In the present study, we simulated the removal of the barn owl's ruff in a virtual acoustic environment. Under normal virtual conditions, differences in azimuthal head-turning behavior between individualized and non-individualized HRTFs were not observed, and the owls were able to discriminate (i.e., they reacted differently to) sounds in the frontal hemisphere and targets in the rear hemisphere, respectively, even if the sounds had equal ITDs. This ability was lost after the virtual removal of the ruff. Elevational head-turn angles were smaller with non-individualized than with individualized HRTFs. The removal of the ruff resulted in a large decrease in elevational head-turn amplitudes. In the following, we shall first discuss the similarities and differences in head-turning between individualized and non-individualized HRTFs, and then turn to the ruffcut situation.

### Sound localization with individualized and non-individualized HRTFs

Our results on the equivalence of the use of individualized and non-individualized HRTFs for azimuthal sound localization in the barn owl are in accordance with findings in for human listeners [22]. Humans use both the ITD of the carrier (up to 1.5 kHz) and the ILD (at frequencies higher than 4 kHz) for azimuthal sound localization (for a review see [23]). In the study by Wenzel et al. [22], azimuthal localization was only marginally impaired when non-individualized HRTFs were used for stimulation. This fact suggests that the small differences in binaural cues between individualized and non-individualized HRTFs were more important for azimuthal sound localization than larger changes in monaural spectral cues.

Likewise, our observation of an increased localization error for large azimuthal or elevational values is similar to what others have observed in the barn owl [3], [6], [7], [9], [12], [24], cat [25], ferret [26], monkeys [27] and humans [28]. Nodal et al. [26] reported that the final head bearing of the ferrets in their study before approaching a free-field sound source rarely exceeded 60° in azimuth, which is well in accordance with our results for the barn owl. Likewise, their observed head-turn latencies of about 200 ms accord with the latencies clustered around 150 ms that we found in our experiments.

The general undershooting, i.e. a too small head-turn angle at peripheral stimulus positions, are a commonly observed phenomenon (see above). It can even occur with free-field sounds, as described by Nodal and coworkers [26] who observed maximum head-turn angles at about ±60° for ferrets even for targets coming from the rear hemisphere. The owls in our study reported all stimuli as being located in the frontal hemisphere, and therefore committed back-front errors under all conditions. However, the owls' azimuthal head-turn angles increased significantly (two-way ANOVA, p<0.001) with increasing stimulus azimuth under stimulation with intact ruff, and the owls localized stimuli differently even if the ITDs at two positions in the rear (140°) and front (40°) were equal (Figure 5).

In a study by Hwang et al. [29] and in the study by Wenzel et al. [22], localization errors under non-individualized conditions increased in the elevational plane compared to localization with individualized HRTFs in humans, whereas azimuthal localization was barely hampered [30]. This is what we found as well. The increased elevational localization errors in humans seem to be related to inter-individual differences in the monaural spectra. This might be so also in the barn owl, and should be tested experimentally in this animal. The spectral notches in the HRTFs of cats [25], [31], rats [32] or owls [5] change most prominently in the central rather than the peripheral field. Therefore, it was not surprising that vertical localization—probably also utilizing frequency-dependent cues—in our owls was better in the frontal than the peripheral field.

It was somehow surprising that the basic features of the localization errors committed by humans and barn owls were similar, although the two species use different cues for sound localization (see [33] for a review). For azimuthal localization errors, this may be due to the fact that ITDs depend mainly on the head diameter and sound source position, but not on frequency (for a review see [16], [23]. This is different for elevational sound localization which utilizes ILDs and monaural spectral cues and where frequency-specific peaks and notches have more influence. The monaural cues underlie stronger individual variations, but this may be overcome by frequency scaling of the directional transfer functions [34]. Kulkarni and Colburn [35] as well as MacPherson and Middlebrooks [36] showed that details of the HRTF spectrum are not as important as their overall shape. This seems also to hold for the barn owl, because the owls were still able to localize the virtual sounds with non-individualized stimuli in the vertical plane, albeit with larger localization errors.

### Localization with simulated ruff removal

In any stimulus condition, the owls reported sound stimuli—including those at stimulus angles >90°—as being in the front. Hence, all owls showed back-to-front reversals. However, under normal conditions, the owls localized targets in the periphery at larger angles than would be expected if the animals were confusing stimulus positions with identical ITDs, and were able to discriminate between targets having the same ITD (Figure 5).

In the ruffcut condition, by contrast, the owls localized targets at ±140° azimuth at a position of about ±40° (Table 2), both positions having the same ITD (Figure 5). They were thus apparently unable to discriminate positions on a cone of confusion, along which ITDs are identical and therefore ambiguous. Given the assumption that ITDs alone are insufficient to distinguish between such ambiguous targets in the rear and front, our findings can only be explained if the barn owls used cues other than ITD for the localization of the stimuli beyond some 110° in azimuth. These cues are not known at the moment. One possibility is that the owls also use ILDs, although we think this is very unlikely, because the owls did not discriminate well between ILDs appearing at large azimuthal values. Monaural spectral cues as observed in the high-frequency range (8–16 kHz) for humans [37] are a more likely candidate, since they allow monaural sound localization in familiar environments [38] and their use to resolve front-back confusions can be specifically trained at least in human listeners [39]. This issue needs to be further investigated in the barn owl.

Discrimination of target positions with the same ITD was not possible after the virtual removal of the ruff (Figure 5). The removal of the ruff changed the distribution of ITDs. After the virtual removal of the ruff, the barn owls behaved as if they exclusively used the information provided by the ITD to compute the amplitude of the head-turn. In other words, ruff removal changed the important additional cues the owl needs to discriminate between positions having the same ITD in the frontal and rear hemispheres. After virtual ruff removal, the bird used the available information from ITDs, but this information was ambiguous and, therefore, the owls could no longer discriminate stimuli with the same ITD, but coming from different hemispheres (see Figure 5). Ruff removal also severely hampered the owl's ability to determine stimulus elevation (Figure 7), which can be explained by the accompanying changes in the ILD distribution [8], [16].

Zahorik et al. [39] reported that human listeners stimulated with non-individualized HRTFs initially had difficulties in localizing sound sources and suffered from front-back reversals. However, their subjects learned to resolve these confusions when they received auditory, visual and vestibular feedback, so that the rate of reversals decreased. The owls in our study did not get feedback on the location of the sound source. This might explain why we did not observe any learning effects. It might be interesting to test whether additional visual feedback might also reduce back-front confusions in the barn owl.

We want to point out that in our case the use of non-individualized HRTFs in general cannot explain back-front confusions as they do in human listeners [22], [39], [40], since back-front reversals occurred in all conditions including individualized stimuli, and were previously observed also with free-field stimuli [6], [26].

Another important question is whether in our experiments the owls could actually perceive the changes in both ITD and ILD that the simulated ruff removal caused. As Figure 1 and Figure S1 demonstrate, the ILD after ruff removal was often larger than the ILD that the owl would perceive naturally. This unnatural experience of ILDs outside the physiological range might result in confusions and hamper the localization ability. However, we think this is unlikely, since it was shown that barn owls can not only process ITDs that are about 5 times larger than the physiologically occurring ITDs [15], but also localize ILDs of up to ±25 dB [7], well outside the natural range. Responses to large ILDs were observed on both the behavioral and neuronal levels [7], although it has to be mentioned that the responses (the neuronal firing rate and the elevational head-turn angles, respectively) in that study reached a saturation plateau at about 10–15 dB ILD, which corresponds approximately to the maximum physiological ILD. Since the high-frequency spatial receptive fields in the auditory cortex of ferrets change depending on the use of individualized versus non-individualized virtual sound stimuli [41], [42], it would be an interesting future project to collect electrophysiological data on the neuronal responses to virtual ruffcut stimuli also in the barn owl.

Stimulation with earphones and varying stimulus parameters like ITD and ILD may be seen as the first step to create a simulated acoustic environment. Such stimulation was used to determine the importance of ITD for azimuthal sound localization [4], [9], [11] and the importance of ILD for elevational sound localization [7], [9], [12]. In humans, presentation of ITD via headphones results in lateralization, but not in externalization [43], [44]. Headphone stimulation removes the specific effects of mainly the pinna on the incoming sound. In barn owls, headphone stimulation without using HRTF-filtered sound signals also removes the effect of the ruff. This is the main effect, since the barn owl has only a small ear flap whose function is unclear.

Thus, the ruff in the barn owl is a structure which is functionally equivalent to the pinna in other animals and humans. Therefore, the virtual removal of the ruff might be expected to result in a similar influence on localization performance as plugging or removal of the pinna of other binaurally hearing species. Cats, for example, use spectral cues in the mid- and high-frequency range for elevational sound localization especially in the median plane [25], [45], [46]. In mammals, the pinna typically increases the monaural gain (in dB) and crucially influences the localization cues [31], [47]. Consequently, the ability to localize sound-source elevation is hampered after removal or occlusion of the pinnae in various species like chinchillas [48], ferrets [49], bats [50], [51] or humans [52]. Thus, our study underlines the functional similarity of the facial ruff in the barn owl with the pinna in mammalian species, including effects on vertical and azimuthal sound localization and specifically in the discrimination of spatial positions with the same ITD.

### Conclusions

Our results show that the facial ruff improves a) peripheral sound localization by increasing the ITD range and by yielding localization cues that allow discrimination of positions with equal ITD, and b) elevational sound localization in the frontal field by introducing a shift of iso-ILD lines out of the midsagittal plane, which causes ILDs to increase with increasing stimulus elevation. We also demonstrate that the changes at the behavioral level might be related to the changes in the binaural physical parameters, ITD and ILD, that occur after the virtual removal of the ruff. The different strengths of the effects on azimuthal and elevational sound localization might be explained by the use of different cues. The functional similarity of the facial ruff with that of the pinna in humans opens the possibility to use these data for autonomous agents [53], improvement of auditory displays [54], [55] or even in hearing aids [56].

## Methods

### Creating a virtual acoustic environment using HRTFs

Three American barn owls (*Tyto alba pratincola*, L.) participated in the behavioral experiments. Care and treatment of the owls was in accordance with the guidelines for animal experiments as approved by the Landespräsidium für Natur, Umwelt und Verbraucherschutz Nordrhein Westfalen, Recklinghausen, Germany, and complied with the NIH Guide for the use and care of laboratory animals. We created a virtual-space environment for the behaving owls (owl H, owl P and owl S). The ruff of these owls was not removed. In this way, they were not impaired in their orienting and social behavior outside experimental sessions, and fixed reference HRTFs could be used for every owl that participated in the behavioral experiment. The owls experienced the changed HRTFs only during the daily experimental session. Therefore, they did not get used to the slightly different spatial sensations of non-individualized HRTFs.

As a reference animal, HRTFs of an anesthetized barn owl (owl 39) were recorded in earlier experiments [16] before and after removal of the ruff feathers. Details of the anesthesia may be found in [57]. The procedure of feather removal, HRTF measurement and calculation of the HRTF filters was previously described in detail [16]. Owl 39 was killed after the procedure. HRTFs from the owls, which participated in the later behavioral experiments (owl H, owl S, owl P), were recorded with normal ruff under anesthesia. All HRTFs were corrected for the influence of the ear canal of the corresponding owl, which differed between the behavioral owls and owl 39. This introduced a systematic error when comparing localization of non-individualized HRTFs, due to the slightly different ear canal. However, comparison of non-individualized HRTFs with and without facial ruff, respectively, considers the influence of the ruff only.

The HRTF measurements were carried out in a sound attenuating chamber (IAC 403A, Industrial Acoustics, Niederkrüchten, Germany). A loudspeaker (MacAudio ML-103E) could be moved along a semicircular track (hoop). The hoop could be rotated on a vertical axis. In effect the loudspeaker could be placed at virtually every spatial position on a sphere of 90 cm diameter from the centre of the owl's head. A microphone (Sennheiser KE4 211-2) with an attached silicone tube was placed 2 mm in front of the eardrum, close enough for precise measurement, but without running the risk of damaging the eardrum [5]. HRTFs were measured at positions from −170° to +160° azimuth and from −70 to +80° elevation with 10° resolution. Negative positions refer to left or downward, respectively.

The recorded transfer functions were corrected for the influence of the hardware components including the microphones and attached tubes by dividing its FFT (fast Fourier transformation) through the FFT of a reference measurement of the microphones alone (without the owl) [16]. For a given HRTF, we performed a cross-correlation of the left and right ear's impulse response to derive the ITD. Broadband ILDs were calculated by subtracting the average level (within the range of 1–12 kHz) of the left from the right ear's impulse response. The resulting ITD and ILD distributions were plotted in a Cartesian coordinate system (Figure S1).

The calculations led to sets of HRTFs for each owl and stimulus condition (normal or removed ruff feathers, respectively) that were stored as finite impulse response filters (FIR) for the right and left ear on a digital controller, HUGO (Institute for Technical Acoustic, Aachen, Germany). Thereby, any incoming signal could be filtered with the HRTFs of a defined spatial position to simulate a free-field sound coming from the corresponding direction (virtual loudspeaker). For stimulation, we generated broadband noise (300–15000 Hz) and converted it to an analogous signal by a TDT DA3-4 digital/analog converter (Tucker-Davis Technologies, Alachua, Florida, USA). A TDT F6 device was included to prevent aliasing.

### Procedure

All behavioral experiments were conducted in the same sound-attenuating chamber that was used for HRTF recording. The owl was placed on a perch in the centre of the chamber in front of a feeder table. The virtual stimuli were presented at the entrance of the ear canal via earphones (Sony MDR-E831LP) after correction for the frequency response of the headphones and the ear canal. The headphone device included the receiver of a real-time tracking system (MiniBird, Ascension Technology Corporation, Burlington, Vermont, USA). Since the headphone device was attached to a metal plate implanted in the owl's skull, it was in a reproducible, fixed position over all experimental sessions. The transmitter detected changes of current flow in a magnetic field induced when the receiver moved within the magnetic field. The corresponding azimuthal and elevational head coordinates reflected the owl's head movements along these axes and were transmitted with 80 Hz sampling rate to the personal computer during the whole experimental session.

During an initial training phase of several months, the owl triggered the next stimulus by fixating a frontal (relative to the owl's natural line of sight) “zeroing window” for some 100 ms. Stimuli consisted of HRTF filtered, static broadband noise with varying length (100–1000 ms). Stimulus positions were selected pseudo-randomly in a range of −140° to +140° in azimuth and −40 to 40° in elevation in steps of 10°. The owl had to turn its head into the correct direction, defined by the azimuthal and elevational components that the stimulus provided, within a target window of ±10° azimuthal and elevational deviation from the target position. If the target window was fixated for at least 150 ms, the owl was automatically rewarded with several hundred milligrams of meat (one day old chicken) from the feeder apparatus. Otherwise, no reward was provided. The training phase continued until the owl showed high performance (>50% of the trials were within the target window).

The actual experimental sessions corresponded to the training procedure, but stimuli had duration of 100 ms and responses were randomly rewarded in 60% of trials. This percentage sufficed to keep the owl motivated for 30–100 trials per day. Individualized HRTFs were presented in 60% of trials, non-individualized HRTFs with intact (20%) and removed ruff (20%) were interspersed in the remaining trials in pseudo-randomized order.

The number of trials, n, depended on the stimulus position. We tested each position in steps of 20° azimuth (from −140° to 140°) at least 18 times. Since the stimulus software determined the HRTF to be used for stimulation depending on the owl's initial head position by rounding to integer steps of 10°, the owl was stimulated in few trials at stimulus positions other than steps of 20° azimuth. We did not include positions with trial numbers smaller than n = 6 into statistical analyses.

### Data analysis

The head movements of the owls were tracked by the MiniBird tracking device (Ascension Technology Corporation, Burlington, Vermont, USA). Head movements with velocities exceeding 20°/s were defined as head-turns. For each trial, we corrected the head-turn track for the owl's initial head-turn position by subtracting the azimuthal head position from the azimuthal component of the track, and the initial elevational head-turn position from the elevational component of the track. This correction allowed us to define the initial head-turn position as 0° azimuth and 0° elevation and enabled comparison of head-turn angles between the individual trials. Figure S2A shows a typical head-turn, segregated into the azimuthal (Figure 2B) and elevational head-turn component (Figure 2C). Azimuthal and elevational components were analyzed separately. The fixation point was defined as that point at which head-turn velocity fell below 20°/s for at least 150 ms. If the owl fixated the target position shorter, or if it had response latencies (the time between trial initiation and onset of the head-turn) of less than 50 ms or more than 500 ms, the trial was counted as an error. Only trials that met the criteria (non-errors) were included in the analysis. For comparison of two data-sample groups (e.g., the fixation points of two different owls at the same stimulus position), we used a two-way ANOVA with Scheffé post-hoc test to reveal dependences of head-turn angles on stimulus parameters and, for further evaluation, a Mann-Whitney test (two-tailed, 95% confidence interval).

For the Boltzman fit to the azimuthal head-turn angles (dependent variable) as shown in Figure 3, we estimated the lower asymptote, A1, and the upper asymptote, A2, from the azimuthal head-turn angles using a nonlinear least squares regression. The Boltzman fit was calculated following the equation
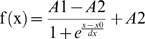
(eq. 1)with x0 as x at y/2 or 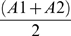 and dx as a time constant.

## Supporting Information

Figure S1 Broadband ITD and ILD distributions. The distribution of ITDs (A–D) and ILDs (E–H) in dependence of the azimuthal (ordinate) and elevational (abscissa) sound-stimulus positions is shown for owl H (A,E) and owl S with intact ruff (B+F), for owl 39 with intact ruff (C,G), and for owl 39 with removed ruff feathers (D,H). Angular values refer to the position in a spherical coordinate system relative to the midsagittal plane (azimuth) and the horizontal plane through the owl's eyes (elevation). Negative azimuthal angles correspond to positions to the left of the owl. Negative elevational angles correspond to positions below the equator. The bold lines indicate the positions where the ITD or ILD are zero, respectively, i.e. the sound reaches the left and the right ear at the same time. The thin black lines connect points with equal ITD (iso-ITD line) in steps of 50 µs or equal ILDs (iso-ILD line) in steps of 2 dB. The maximum negative ITD and the maximum negative ILD are marked with a “-” sign, whereas the maximum positive ITD and ILD are marked with a “+” sign. The angular position of the extrema is given above each panel, together with the corresponding ITD (in µs) and ILD (in dB), respectively.
(5.15 MB EPS)

Figure S2 Head-turn movement. (A) A typical head-turn movement tracked with the MiniBird device is shown for a trial with owl H. The stimulus position was 100° azimuth and 0° elevation (arrows). The owl's initial head position was defined at 0° azimuth and 0° elevation on a head-centered coordinate system. The owl's final fixation position (head-turn velocity <20°/s) is marked by the circle. The owl then turned its head back to the feeder device (close to 0° azimuth and −50° elevation). (B) The azimuthal head-turn component of the trial shown in A) is plotted on a linear time scale. Stimulus duration (100 ms) is indicated by the bold black line. The head-turn started at the position marked by the circle. The owl fixated the azimuthal position marked by the triangles (defined as the head-turn angle) before turning back to the position of the feeder. (C) As in (B), the elevational head-turn component is plotted against time (in ms). The dotted lines delineate the time points between about 380 and 800 ms and mark the elevational gaze direction during target fixation.
(0.90 MB EPS)

Figure S3 Latencies. (A–C) owl H, (D–F) owl S. The diameters of the black circles represent median reaction times in response to stimulation with individualized HRTFs (A,D), normal HRTFs of owl 39 (B,E) and HRTFs of owl 39 with removed facial ruff (C,F) at the three stimulus elevations. Gray numbers indicate significant deviation (Mann-Whitney test, p<0.05) of the latency from that measured at the corresponding stimulation site with individualized HRTFs. No systematic change of the median reaction times occurred when the owls were stimulated with non-individualized HRTFs.
(2.32 MB EPS)

Figure S4 Localization errors. The difference between the azimuthal stimulus angle and the corresponding head-turn angle in degree was defined as the localization error. For each stimulus elevation (rows) and owl (columns, A–C: owl H; D–F: owl S; G–I: owl P), localization errors (mean±SD) are plotted as a function of stimulus azimuth (black lines = individualized HRTFs for owls H and S respectively owl H's HRTFs for owl P, blue = owl 39 normal, red = owl 39 ruffcut). Positive localization errors indicate that the owl fixated a position too close to zero degree (“undershooting”), negative angles indicate overshooting. Positive localization errors gradually increased with increasing stimulus azimuth. Distributions hardly varied between individualized HRTFs and HRTFs of owl 39 normal (significant differences found with a Mann-Whitney test, two-tailed, are marked with blue asterisks), but stimulation with HRTFs of owl 39 with removed ruff resulted in a significantly larger localization error (marked with red asterisks) especially in the peripheral field (±140°, see also Table 2). All data points are shown to give a better picture of the owls' behavior. Statistical comparisons were only performed for data points including at least n = 3 trials. Blue asterisks mark positions with significant differences (Mann-Whitney test, p<0.05) between individualized HRTFs (owl P: owl H's HRTFs) and owl 39 normal, respectively owl 39 ruffcut (red asterisks)).
(1.98 MB EPS)

Figure S5 Azimuthal head-turn angles at varying elevations. For owl H (individualized HRTFs), the azimuthal head-turn angles in degree (mean±SD) for stimulus positions with at least n = 10 trials are plotted against the azimuthal stimulus position in degree. Localization of the exact stimulus position would result in a line with a slope of 1 (black line). The curved lines represent Boltzman fits to the azimuthal head-turn angles (black circles: 0° stimulus elevation; blue circles: 40° elevation). Angles at 40° stimulus elevation were significantly (Mann-Whitney test, *p<0.05, **p<0.01, ***p<0.001) smaller than at 0° elevation. This held also for -40° stimulus azimuth (data not shown for better clarity) and for the other owls (data not shown). Each data point includes 10 to 28 trials (n).
(1.36 MB EPS)
